# Expert consensus on the minimum clinical standards of practice for Nigerian physiotherapists working in intensive care units: A modified Delphi study

**DOI:** 10.7196/AJTCCM.2021.v27i3.137

**Published:** 2021-10-04

**Authors:** I Idris, A W Awotidebe, N B Mukhtar, R N Ativie, J M Nuhu, I C Muhammad, A S Danbatta, R A Adedoyin, J Mohammed

**Affiliations:** 1 Department of Physiotherapy, General Hospital Gombe, Gombe, Nigeria; 2 Department of Physiotherapy, Faculty of Allied Health Sciences, Bayero University Kano, Kano, Nigeria; 3 Department of Medical Rehabilitation, University of Nigeria, Nsukka, Nigeria; 4 Department of Physiotherapy, Usmanu Danfodiyo University Teaching Hospital, Sokoto, Nigeria; 5 Department of Physiotherapy, Aminu Kano Teaching Hospital, Kano, Nigeria; 6 Department of Medical Rehabilitation, Obafemi Awolowo University, Ile-Ife, Nigeria

**Keywords:** expert consensus, critical care physiotherapy, Delphi technique, standards of practice

## Abstract

**Background:**

Management of patients in intensive care units (ICUs) needs staff with a recommended level of expertise and experience owing to the life-threatening nature of illnesses, injuries and complications that these patients present with. There are no specific guidelines governing physiotherapy practice in ICUs in Nigeria. Hence, there is a need to have expert consensus on the minimum clinical standard of practice for physiotherapists working in ICUs as a first step to proposing/developing guidelines in the future.

**Objectives:**

To assess the expert consensus on the minimum clinical standard of practice for physiotherapists working in ICUs in Nigeria.

**Methods:**

Physiotherapists with working experience in Nigerian ICUs were purposively recruited into the present study using a modified Delphi technique. A questionnaire comprising 222 question items on the role of physiotherapy in critical care was adopted and administered to the participants over three rounds of Delphi procedure (online). Participants checked either ‘essential’, ‘not essential’ or ‘unsure’ for each question item. For each question item to be considered ‘essential’ or ‘not essential’, a consensus agreement ≥70% had to be met. Questions without consensus were further modified by providing definition or clarification and presented in subsequent rounds. Data were analysed descriptively.

**Results:**

We recruited 26 expert physiotherapists who consented to the study and completed the first round of the study. The majority of the physiotherapists (*n*=24) remained in the study after the third round. A total of 178 question items were adjudged to be ‘essential’ after the first round, and a further 15 and three additional items were subsequently adjudged to be as ‘essential’ after modifying the outstanding question items during the second and third rounds, respectively. No consensus was reached for 24 items. None of the question items were ranked as ‘not essential’ after all the rounds.

**Conclusion:**

Expert consensus was achieved for a substantial number of question items regarding knowledge and skills for assessment, condition and treatment items of the questionnaire by experienced critical care physiotherapists in Nigeria.

## Background

Intensive care units (ICUs) are specially staffed and equipped hospital wards for management of patients with life-threatening illnesses, injuries or complications. However, the range to which different hospitals provide services to critically ill patients depends on the skills, expertise, facilities and clinical specialties available in the hospitals.^[1]^ Physiotherapy is one of the fundamental interventions administered to patients in ICU.^[2]^ The major goals of physiotherapy in the ICU include maintaining/restoring the general patient’s functional capacity, and restoring respiratory and physical independence. Physiotherapy also helps to decrease the risks associated with stay in the ICU such as acquired muscle weakness, physical deconditioning and poor quality of life.^[3]^ Moreover, the positive impact of physiotherapy in the management of patients whose conditions require critical care is well documented^[4,5]^ and noted to improve survival rates.^[6]^ There is moderate-to-strong evidence to support the role of physiotherapy for managing critically ill patients.^[7]^

The ICUs in resource-restricted settings have limited infrastructure, materials and human resources.^[8]^ In the UK, just as in other developed countries, physiotherapy is provided for 24 hours/day and 7 days a week (including on-call and public holidays) for patients in the ICUs.^[7]^ In Nigeria, ICU patients are managed by physiotherapists every day of the week, but no evidence exists to support whether this is actually instituted in the ICUs standard of practice or guidelines. Consequently, some fresh graduate physiotherapists may start their first on-call service with less or no previous hands-on experience or training in managing critically ill patients in some ICUs.^[9]^ Some centres compensate for this inadequacy by organising in-house/local critical care programmes/ICU workshops or mentoring to solve the problems of novice physiotherapists working in the ICU. Nevertheless, a bachelor’s degree remains the least requirement for physiotherapists to work in ICUs in Nigeria. In short, the quality of care provided in the ICU largely depends on the skills of the attending physiotherapist.

Previous modified Delphi studies by Skinner *et al*.^[10]^ in Australia, Twose *et al*.^[11]^ in the UK and Takahashi *et al* ^[12]^ in Japan identified 132, 107 and 199, respectively, question items that are considered ‘essential’ for physiotherapists working in critical care units, and form a 222-item questionnaire developed by Skinner *et al*.^[10]^ A similar study using the same questionnaire is necessary in Nigeria to identify and possibly suggest ways to standardise the competencies of physiotherapy practice in ICUs. As no standards of practice currently exist for the training of physiotherapists working in critical care in Nigeria and adopting an existing questionnaire has precedence in Delphi methodology.^[13]^ Using the questionnaire developed by Skinner *et al*. ^[10]^ offered us the opportunity to contextualise our findings using a global point of view. Moreover, ICU patients require the best care possible, irrespective of the setting. The absence of national treatment guidelines indicates that most ICU interventions in resource-limited settings are often based upon treatment guidelines adopted from developed countries or international stakeholders.

The development of critical care in resource-poor settings relies on service improvements, including leveraging human resources through training, a focus on sustainable technology, continuous analyses of cost effectiveness and sharing of context-specific best practices.^[14]^ Therefore, the present study is designed to explore the consensus of experienced physiotherapists regarding the minimum clinical standards of practice that physiotherapists working in critical care in our environment should possess. Moreover, the findings from this study could help in focusing future treatment guidelines, postgraduate education and ICU-related training of critical care physiotherapists in Nigeria and other similar countries.

## Method

The present study followed a guide for Conducting and REporting of DElphi Studies (CREDES).^[15]^

### Ethics

Ethical approval was sought and obtained from the Research and Ethical Committee of the College of Health Sciences, Bayero University, Kano (ref. no. NHREC/06/12/19/22). The ethical protocols of the Declaration of Helsinki including the ethical principles of informed consent, privacy and confidentiality of data provided were followed in the Delphi rounds.

### Design

A modified Delphi technique was used to seek consensus on the minimum standards of practice for physiotherapists working in ICUs in Nigeria. Delphi techniques using online surveys are easily accessible and were the most appropriate for this study because they allow participants from across Nigeria to take part in the study from start to finish.^[16,17]^ In addition, the Delphi technique allows for formation of consensus or exploration of a field beyond existing knowledge. It can be adapted to the particular requirements of the research question, and it takes the form of open and exploratory questions to standardised confirmatory approaches.^[15]^ In the present study, the basic Delphi technique was modified to allow for question clarification, addition of new items and eventual agreement of the items by participants. Furthermore, in the second and third rounds of the Delphi study, additional definitions with examples (where possible) were provided for some terminologies where consensus was not reached in the first round to further enhance understanding of the terms.

### Respondents

The prospective participants for this study were recruited using purposive sampling, alongside a snowballing method via advertisement through the official communication channels of the physiotherapy practice regulatory board and professional associations in Nigeria: namely (i) Medical Rehabilitation Therapist Registration Board of Nigeria (MRTB); (ii) Nigeria Society of Physiotherapy; and (iii) Association of Clinical and Academic Physiotherapists of Nigeria. Participants’ recruitment was also made via social media platforms, particularly WhatsApp and Facebook groups of professional associations (e.g. Nigerian physiotherapists). Eligibility criteria were only communicated to participants after they had accepted an invitation to participate in the present study.

Sample size was not fixed so that we would be able to recruit as many participants as possible who met the eligibility criteria. The eligibility criteria for participation included the following: (i) physiotherapists with a minimum of 5 years’ working experience, three of which must be in a senior role within the critical care setting; (ii) physiotherapists involved in the supervision or teaching of physiotherapy staff working on-call or completing emergency duty; and (iii) academic physiotherapy staff involved in the provision of entry-level cardio-respiratory physiotherapy with at least two articles published in the area of critical care.

### Questionnaire

The questionnaire utilised in the present study was adopted from Skinner *et al*.^[10]^ The questionnaire was structured and planned content-wise to be as extensive as possible across the physiotherapy role in critical care.^[10]^ We did not pilot the questionnaire because it had been previously used in multiple studies ^[11]^ and we also aimed to contextualise the opinions of experienced physiotherapists in comparison with those in previous studies. Twose *et al*.^[11]^ also adopted and used the same questionnaire without piloting. The questionnaire further highlighted that the motive of the present study was to determine the minimum standard of clinical practice that should be expected from physiotherapists to qualify them to work autonomously and safely with patients in critical care settings. The questionnaire consisted of 222 question items. For each question item, participants were asked to either check ‘essential’, ‘not essential’, or ‘unsure’ option. Respondents were also asked to submit additional items that were not previously included if they thought it necessary and essential for inclusion in the first round.

### Procedure

Three rounds of questionnaire administration were sent to the study participants between 10 August 2020 and 2 October 2020, with each round lasting an average of 2 weeks. Delbecq *et al*.^[18]^ recommended that 2 weeks is enough for Delphi participants to attempt each round. The study questionnaires were administered using Google forms via WhatsApp or email. The participants’ information sheets were included in the invitation message. Reminders (phone calls, SMS and WhatsApp messages) were also sent to non-responders 7 days and a day before the deadline. Participants were also assured of the anonymity of all information provided so that they would not be afraid to admit their knowledge or lack of knowledge on any question item. Moreover, the participants were not asked to provide their names or any identifiers that might link them to any information provided. Demographic variables were only collected during round one, and were analysed separately by a blinded author (ASD). On completion of each round, participants were sent a personalised message thanking them and requesting for their co-operation in the subsequent rounds.

### Data analysis

Data were analysed using descriptive statistics. Since demographic data were collected in the first round only, the data were summarised as a mean with standard deviation (SD), frequencies and percentages using Microsoft Excel. For each question item, a consensus to determine if such a question item was either ‘essential’ or ‘not essential’ was based on a consensus agreement of ≥70%. A study by the original developers of the instrument recommended a threshold of 70%.^[10]^ Therefore, this threshold was used in the present study to allow for comparison of the study findings later. The percentage of ‘unsure’ responses was also taken into consideration to determine whether consensus was reached. Specifically, consensus for each item was calculated by subtracting the number of ‘unsure’ responses from the percentage of ‘essential’ responses. For example, if an item ranked 74% as ‘essential’ and the percentage of ‘unsure’ responses was 9%, then the percentage of ‘unsure’ responses was removed from that of ‘essential’. For any item to be considered as ‘essential’ or ‘not essential’, consensus had to be >70% in any of the three rounds.

## Results

**Fig. 1:**
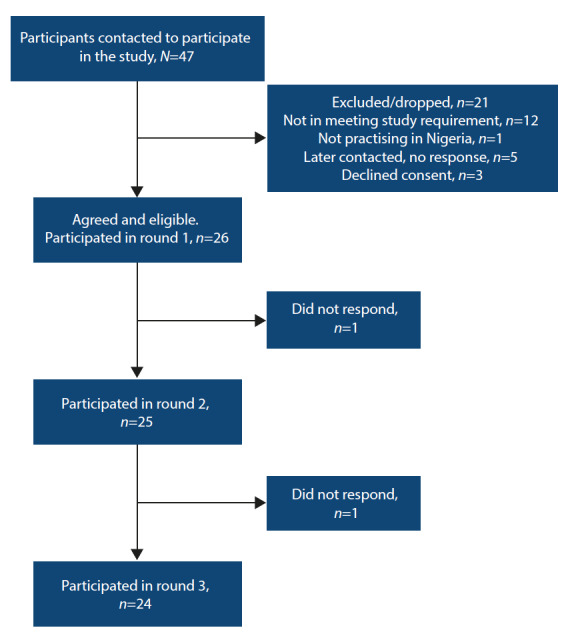
Flowchart of the recruitment and Delphi process.

A panel of 47 experts (experienced physiotherapists working in Nigerian ICUs) responded to our invitation to participate in the Delphi study, and 32 potential participants met the inclusion criteria and were invited to participate in the first round of the present study. However, 81.3% (*n*=26) of the participants completed the consent forms and participated in the first round of the present study. Three-quarters of the participants (75%; *n*=24) remained by the end of the third round (Fig. 1).

**Fig. 2:**
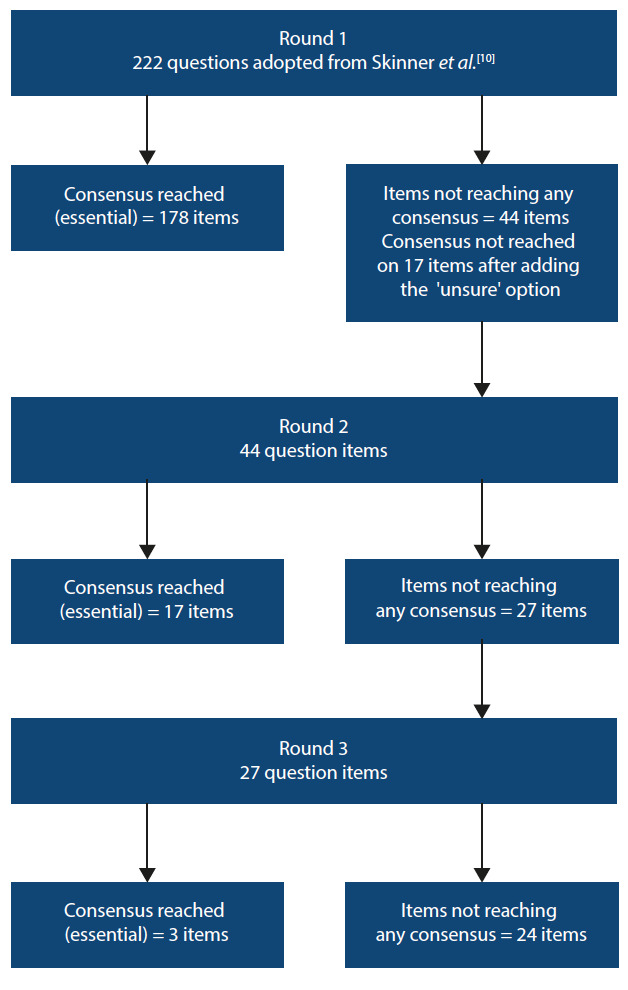
Flow of items through the three rounds of the Delphi process.

The participants comprised physiotherapy clinicians (*n*=24) and academics (*n*=2). The participants were drawn from all the six geopolitical regions of Nigeria (North East (*n*=4), North Central (*n*=5), North West (*n*=9), South East (*n*=4), South-South (*n*=1) and South West (*n*=3)). The majority of the participants (61.5%) had 10 years’ or more ICU work-related experience. A substantial number of them (*n*=19) were staff of federal tertiary hospitals (Table 1).

**Table 1:** Demographics of the experts that participated in the Delphi study (*n*=26)

**Characteristics**	Participants, *n *(%)*
**Age (years), mean (SD)**	40.8 (8.6)
**Gender**	
Male	16 (61.5)
Female	10 (38.5)
**Institution of practice**	
University hospitals	18 (69.2)
University academics	2 (7.7)
Medical centre	1 (3.8)
Specialist hospitals	3 (11.6)
Private hospitals	2 (7.7)
**Years of experience in ICU**	
5 - 10	10 (38.5)
10 - 15	5 (19.2)
15 - 20	6 (23.1)
>20	5 (19.2)
**Published article**	
<2	17 (85.0)
2 - 6	3 (15.0)

ICU = intensive care unit

SD = standard deviation

*Unless otherwise specified

The first round of the study consisted of 222 items, of which 178 were considered as ‘essential’ by the respondents. One additional item was suggested by one participant; however, it was not added in the second round because it was a duplicate of an existing question item. None of the question items qualified to be ranked as ‘not essential’ in the first round, and consensus was not reached on 17 items on account of ‘unsure’ responses. In the end, the remaining 44 items were presented again in the second round.

Following the second round, 17 additional items were ranked as ‘essential’ and consensus was reached for 27 items. Still, no item was ranked as ‘not essential’, and no additional item was suggested by the participants in the second round. The remaining 27 question items were further presented in the third round. Consensus was only reached for 3 question items that were categorised as ‘essential’. Consequently, consensus was not reached for the remaining 24 items. Just like in rounds 1 and 2, no question item was ranked as ‘not essential’ in the third round.

Overall, the participants reached consensus on 197 items as ‘essential’ after three rounds. While no items were considered ‘not essential’, no consensus was reached for 24 items. Detailed breakdown of the question items that reached consensus following the Delphi rounds are presented in Tables 2, 3 and 4. The question items that did not reach consensus after the third round are presented in Table 5.

**Table 2:** Assessment items determined as essentials (consensus >70% ‘essential’)

****	**Round 1**	**Round 2**	**Round 3**
**As a minimum standard a physiotherapist can accurately interpret readings from clinical monitoring including:**
Body temperature	100
Heart rate	100
Blood pressure	100
Basic ECGs, SpO_2_/pulse oximetry	100
End tidal carbon dioxide	96.2
Fluid intake and output	100
**As a minimum standard a physiotherapist can understand equipment (including recognition of equipment) and understand the implications for physiotherapy of:**
Oxygen therapy devices	100
Endotracheal tubes and tracheostomy	92.3
Central venous catheters	88.5
Arterial lines	96.2
Venous blood gas interpretation (including SvO_2_) Vascath/haemodialysis catheter/continuous veno-venous.	61.5^†^	88
Intercostal catheters	84.6
Wound drains	80.8
Indwelling urinary catheter	100
Nasogastric tubes	100
**As a minimum standard a physiotherapist can accurately interpret findings from laboratory investigations including:**
Haemoglobin	100
Platelets, APTT, INR	92.3
White cell count	88.5
Blood glucose levels	100
**As a minimum standard a physiotherapist is aware of the actions and implications for physiotherapy of the following medications:**
Vasopressors/inotropes	84.6
Basic electrolytes	100
Anti-hypertensives	92.3
Anti-arrhythmia	100
Sedation and neuromuscular paralysing agents	61.5*	92
Bronchodilators	92.3
Mucolytics	69.3*	92
**As a minimum standard a physiotherapist can independently interpret findings from imaging investigations (excluding the imaging report) including:**
Chest radiographs	96.2
**As a minimum standard a physiotherapist can interpret the results from neurological equipment/examinations and functional tests including:**
Intra-cranial and cerebral perfusion pressure monitors	96.2
An ability to interpret an assessment of sedation levels (e.g. Ramsey Sedation Scale, Riker, Richmond-Agitation Sedation Scale)	84.6
An ability to perform a neurological examination of motor and sensory functions (e.g. light touch, pain) e.g. ASIA score	100
An ability to interpret a Glasgow Coma Score	100
**As a minimum standard a physiotherapist can perform and accurately interpret the results of common respiratory examinations including:**
Observation of respiratory rate	100
Patterns of breathing	96.2
Palpate the chest wall	100
Auscultation	100
**As a minimum standard a physiotherapist understands the key principles of providing the following differing modes of mechanical/assisted ventilation including:**
CPAP	92.3
PEEP/EPAP	96.2
SIMV (volume)/(pressure)	69.2*	92
BiLevel	46.2*	88
PS/IPAP	92.3
**As a minimum standard a physiotherapist can assess and interpret mechanical ventilation settings/measurements including:**
Respiratory rate	96
Peak inspiratory pressure	92.3
Inspiration: expiration ratio	100
Tidal volume	100
Breath types (spontaneous, mandatory, assisted)	100
The levels of FiO_2_	100
The levels of PEEP	100
The levels of PS	88.5
**As a minimum standard a physiotherapist can:**
Assess the effectiveness/quality of a patient’s cough	100
Record and interpret observations from physical clinical examination
**As a minimum standard a physiotherapist can interpret indices from blood-gas measurement including:**
pH	100
PaCO_2_	100
PaO_2_, SpO_2_, SaO_2_	100
HCO_3_	100
Base excess	92.3
P_50_	65.4^†^	92
**A physiotherapist can complete musculoskeletal and/or functional assessments including:**
Manual muscle testing	69.2*	84
Range of motion	84.6
Deep-vein thrombosis screening	100
Peripheral oedema	92.3
**As a minimum standard a physiotherapist can understand equipment (including recognition of equipment) and understands the implications for physiotherapy of:**
Extra-corporeal membrane oxygenation	69.2*	80
Intracranial pressure monitors and extra-ventricular drains	96.2
**As a minimum standard a physiotherapist can accurately interpret readings from clinical monitoring including:**
Advanced ECGs	80.8
Nutritional status including feed administration, volume and type	61.5*	100
**As a minimum standard a physiotherapist can accurately interpret findings from laboratory investigations including:**
Haematocrit	96.2
Creatinine kinase	96.2
Neutrophil count	92.3
Albumin	92.3
Liver function tests	88.5
**As a minimum standard a physiotherapist is aware of the actions and implications for physiotherapy of the following medications:**
Calcium channel blockers, cerebral diuretics, hypertonic saline	96.2
Nitric oxide	92.3
**As a minimum standard a physiotherapist can independently interpret findings from imaging investigations (excluding the imaging report) including:**
Skeletal X-rays	96.2
CT – Brain	100
CT – Chest	100
CT – Spine	100
MRI – Brain	100
MRI – Spine	96.2
MRI – Chest	100
Ultrasound – Chest	96.2
**As a minimum standard a physiotherapist can interpret the results from neurological equipment/examinations and functional tests including:**
Electroencephalograms	88.5
An ability to perform a Glasgow Coma Score	100
An ability to perform an assessment of sedation levels	100
An ability to interpret an assessment of cranial nerve function	96.2
**As a minimum standard a physiotherapist understands the key principles of providing the following differing modes of mechanical/assisted ventilation including:**
High frequency oscillatory ventilation	88.5
**As a minimum standard, a physiotherapist can assess and interpret mechanical ventilation settings/measurements including:**
Static and/or dynamic lung compliance measurements	92.4
Upper and lower inflection points of P-V curves	92.4
Maximum inspiratory pressure measurements	92.4
Maximum expiratory pressure measurements	88.5
**As a minimum standard a physiotherapist can:**
Assess the effectiveness/quality of a patient’s cough Record and interpret observations from physical clinical examination	100
Perform respiratory function tests (e.g. for measurements of FEV1, FVC, PEF)	100
Perform and interpret percussion note	96.2
Measure peak cough flow on or off mechanical ventilation	84.6
Measure peak inspiratory flow rate: Peak Expiratory Flow	80.8
Perform a spontaneous breathing trial	96
Interpret the rapid shallow breathing index	80.8
Perform a swallow assessment	84.6
**As a minimum standard a physiotherapist can interpret indices from blood gas measurement including:**
PaO_2_/FiO_2_ ratio	100
A-a gradient	61.6*	96
Oxygen content (CaO_2_)	88.5
Venous blood gas interpretation (including SvO_2_)	69.2*	88
**A physiotherapist can complete musculoskeletal and/or functional assessments including:**
Dynamometry	88.5
Objective measures of physical function	100
Perform and Interpret Chelsea Critical Care Physical Assessment Tool	92.3
Objective measures of cardiopulmonary exercise tolerance	100
Objective measures of quality of life	84.6
**As a minimum standard a physiotherapist can provide the following techniques, including an understanding of indication, contraindications, evidence for technique and progressions:**
Positive pressure devices for airway clearance (e.g. AstraPEP, PariPEP, TheraPEP, or oscillating expiratory pressure devices like Acapella, Flutter)	96.2
Periodic/intermittent CPAP (non-invasive via mask) including initiation and titration of NIV/BiPAP – for Type I or Type II respiratory failure, initiation and titration of e.g. COPD exacerbation with hypercapnia	92.3
NIV/BiPAP – intermittent, short term applications during physiotherapy to assist secretion mobilisation techniques or lung recruitment including initiation and titration of assisted coughing - subcostal thrusts for spinal cord injuries	57.7*	84
Ventilator hyperinflation via an endotracheal tube or tracheostomy	46.2*	92
**As a minimum standard a physiotherapist can appropriately request/coordinate the following**
Titration of inotropes to achieve physiotherapy goals	82.6
**As a minimum standard a physiotherapist is aware:**
Of key literature that guides evidence-based physiotherapy practice in critical care settings	96.2
**As a minimum standard a physiotherapist can accurately interpret readings from clinical monitoring including:**
Central venous pressure	100
**As a minimum standard a physiotherapist can accurately interpret findings from laboratory investigations including:**
Renal function tests e.g. urea and creatinine	100
Sputum cultures	96.2
**As a minimum standard a physiotherapist can**
Determine the appropriateness of a patient for extubation	82.6
Determine the appropriateness of a patient for tracheostomy decannulation	82.6

ECG = electrocardiogram; SpO_2_ = oxygen saturation; SvO_2_ = mixed venous oxygen saturation

APTT = activated partial thromboplastin time; INR = international normalised ratio; CPAP = continuous positive airway pressure

PEEP/EPAP = positive end-expiratory pressure; SIMV = synchronised intermittent mandatory ventilation; PS = pressure support

IPAP = inspiratory positive airway pressure; FiO_2_ = fraction of inspired oxygen; PaCO_2_ = partial pressure of CO_2_

PaO_2_ = partial pressure of O_2_; HCO_3_ = bicarbonate; P_50_ = oxygen tension at which haemoglobin is 50% saturated

CT = computed tomography; MRI = magnetic resonance imaging; FEV1 = forced expiratory volume in one second

FVC = forced vital capacity; PEF = peak expiratory flow

*Consensus not reached (>70%) after considering the scored of ‘unsure’

^†^Consensus not reached (>70%)

**Table 3:** Condition items determined as ‘essential’ (consensus >70%)

****	**Round 1**	**Round 2**	**Round 3**
**As a minimum standard a physiotherapist understands pathophysiology and presenting features, likely medical management and implications for physiotherapy for a range of conditions including:**
Respiratory failure types I and II	100
Community acquired/nosocomial/hospital-acquired pneumonia	100
Pleural effusion	100
Obstructive respiratory disease	100
Restrictive respiratory disease	100
Suppurative lung diseases	96.2
Acute lung injury/acute respiratory distress syndrome	100
Acute coronary syndrome	96.2
Shock (cardiogenic)	100
Heart failure	100
Post-abdominal surgery	96.2
Renal failure: acute and chronic	96.2
Immunocompromise	92.3
Systemic inflammatory response syndrome	96.2
Shock (septic)	100
Multi-organ failure	100
ICU-acquired weakness	100
Guillain-Barre Syndrome	68.2*	68	87.5
Thromboembolic disease	96.2
Intracerebral haemorrhage/Subarachnoid haemorrhage	100
Traumatic brain injury	100
Chest trauma	100
Spinal cord injury	96.2
Neuromuscular disease	96.2
**As a minimum standard a physiotherapist understands pathophysiology and presenting features, likely medical management and implications for physiotherapy for a range of conditions including:**
Post-cardiac surgery	100
Post-thoracic surgery	100
Pancreatitis	88.5
Metabolic/electrolyte disturbances	96.2
Fat embolism	88.5
Brain death and organ procurement	76.9
Multi-trauma	96.2
Sleep-disordered breathing (e.g. obstructive sleep apnoea, hypoventilation)	88.5
**As a minimum standard a physiotherapist can determine the appropriateness of a patient for:**
extubation	82.6
tracheostomy decannulation	82.6
**As a minimum standard a physiotherapist understands pathophysiology and presenting features, likely medical management and implications for physiotherapy for a range of conditions including:**
Hepatitis	69.3*	88
Organ transplantation	92.3
Burns	100

*Consensus not reached (>70%) after considering the scored of ‘unsure.’

**Table 4:** Treatment items determined as ‘essential’ (consensus >70%)

****	**Round 1**	**Round 2**	**Round 3**
**As a minimum standard a physiotherapist can provide the following techniques, including an understanding of indications, contraindications, evidence for the technique and progressions:**
Oxygen therapy including initiation and titration of oxygen therapy	92.3
Humidification	88.5
Active cycle of breathing technique	96.2
Manual airway clearance techniques – percussion, vibration, chest shaking	100
Intermittent positive pressure breathing	96.2
Mechanical insufflation-exsufflation	84.6
Supported coughing	92.3
Directed coughing/instructing the patient to cough effectively	96.2
Assisted coughing – chest wall	96.2
Cough stimulation – oropharyngeal catheter stimulation	96.2
Manual hyperinflation via an endotracheal tube or tracheostomy	92.3
Nasopharyngeal airway suctioning, including insertion of NP airway	96.2
Oropharyngeal airway suctioning, including insertion of OP airway	88.5
Suction via a tracheal tube (ETT, tracheostomy, mini-tracheostomy)	100
Instillation of normal saline into the endotracheal tube	88.5
Patient positioning for respiratory care – including use of side lie, sitting upright, postural drainage (modified or head down tilt)	100
Patient positioning for prevention of pressure ulcers, management of tone, maintenance of musculoskeletal function	100
Mobilisation of non-ventilated patient	100
Mobilisation of ventilated patient	96.2
Bed exercises	96.2
Nasal high flow	88.5
Feldenkreis	61.5^†^	68	87.5
**As a minimum standard a physiotherapist can appropriately request/coordinate the following:**
Titration of analgesia to achieve physiotherapy goals	53.8*	68	82.6
**As a minimum standard a physiotherapist understands the key principles of providing the following differing modes of mechanical/assisted ventilation including:**
Assist-control	100
Airway pressure release ventilation	96.2
Weaning protocols	100
**As a minimum standard a physiotherapist can:**
Interpret respiratory function tests (e.g. for measurements of FEV1, FVC, PEF)	100
**As a minimum standard a physiotherapist can interpret indices from blood gas measurement including:**
Lactate	96.2
**As a minimum standard a physiotherapist has knowledge of methods for advanced haemodynamic monitoring, can interpret the measurements and understands the implication of these for physiotherapists:**
Implanted or external pacemakers and determine presence of pacing on ECG	92.3
**A physiotherapist can complete musculoskeletal and/or functional assessments including:**
Ability to assess tone (e.g. utilising a modified Ashworth scale) and reflexes FEV1; FVC; PEF	96.2
**As a minimum standard a physiotherapist can provide the following techniques, including an understanding of indications, contraindications, evidence for the technique and progressions:**
Glottal stacking (frog breathing)	46.2*	100
Other breathing techniques	100
Autogenic drainage	88.5
NIV/BiPAP - for use during exercise or mobilisation including initiation and titration	60*	84
Cough stimulation - tracheal rub	96.2
Recruitment maneuvers, e.g. staircase	92.3
Bronchial lavage	80.8
Assisting bronchoscopy via delivery of secretion	88.5
Mobilisation techniques during the procedure	96.2
Patient prone positioning in severe respiratory	84.6
Failure/acute lung injury	96.2
Inspiratory muscle training	100
Splinting and/or casting for the upper limbs and lower limbs	100
Collars	92.3
Braces	96.2
Treadmill, cycle ergometry or stationary bike, additional rehabilitation techniques (e.g. hydrotherapy, Wii)	96.2
**As a minimum standard a physiotherapist can:**
Non-invasive ventilation	69.3*	92

FEV1 = forced expiratory volume in one second

FVC = forced vital capacity

PEF = peak expiratory flow

*Consensus not reached (>70%) after considering the scored of ‘unsure’

^†^Consensus not reached (>70%)

**Table 5:** Items not reaching any consensus

****	**Round 1**	**Round 2**	**Round 3**
**As a minimum standard a physiotherapist can understand equipment (including recognition of equipment), understand the implications for physiotherapy of:**
Haemofiltration	61.5*	64	66.7
Intra-aortic balloon pump	69.2^†^	64	66.7
Sengstaken-Blakemore/Minnesota tubes	60^†^	48	50
**As a minimum standard a physiotherapist can interpret indices from blood gas measurement including:**
Anion gap	50^†^	60	62.5*
**As a minimum standard a physiotherapist has knowledge of methods for advanced haemodynamic monitoring, can interpret the measurements and understands the implication of these for physiotherapists:**
Pulmonary arterial catheter measurements	69.2^†^	70.8*	66.7*
PiCCO measurements	50^†^	50	62.5*
**As a minimum standard a physiotherapist can accurately interpret findings from laboratory investigations including:**
Troponin	53.9*	66.7	66.7*
C-reactive protein	63.7*	68	66.7
Procalcitonin	57.7*	68	66.7
**As a minimum standard a physiotherapist is aware of the actions and implications for physiotherapy of the following medications:**
Prostacyclin (PG12)	57.1*	60	62.5*
**As a minimum standard a physiotherapist can interpret the results from neurological equipment/examinations and functional tests including:**
Ability to perform a delirium assessment	65.4*	52	58.3
**A physiotherapist can complete musculoskeletal and/or functional assessments including**
Bioimpedence testing of body composition	65.4*	56	66.7
**As a minimum standard a physiotherapist understands pathophysiology and presenting features, likely medical management and implications for physiotherapy for a range of conditions including:**
Pancreatitis	60*	52	56.5
**As a minimum standard a physiotherapist can:**
Perform a cuff volume and/or pressure test on an endotracheal tube or tracheostomy	61.5^†^	48	45.8
**As a minimum standard a physiotherapist can provide the following techniques, including an understanding of indications, contraindications, evidence for the technique and progressions:**
Performing bronchoscopy independently	57.7	44	58.3
**As a minimum standard physiotherapist can:**
Intubate a patient	57.7^†^	48	50
Extubate a patient	65.4^†^	64	54.2
Lead the co-ordination of weaning protocols	61.5^†^	60	58.3
Lead the co-ordination of cuff deflation trials	48^†^	48	45.8
Lead the co-ordination of speaking valve trials	50^†^	52	45.8
Determine the appropriateness of a patient for tracheostomy decannulation	50^†^	56	47.8
Decannulate a tracheostomy	50^†^	44	54.2
Tracheostomy exchange	46.2^†^	48	54.2
**As a minimum standard a physiotherapist can appropriately request/coordinate the following:**
Titration of sedation to achieve physiotherapy goals	46.1*	64	66.6

*Consensus not reached (>70%) after considering the scored of ‘unsure’

^†^Consensus not reached (>70%)

## Discussion

The present study aimed to assess the consensus of experienced physiotherapists working in ICUs in Nigeria with the view of determining a consensus for minimum standards of clinical practice. The present study is important in our environment because of the growing concerns about the variability of skills, level of qualifications, postgraduate experiences and clinical practice acumen of physiotherapists treating critically ill patients. Earlier studies highlighted varying standards of education, changing the on-call services, reduction in workforce capacity and irregularity in staff entry level.^[19,20]^ We considered physiotherapists with ≥5 years of work experience in critical care settings or emergency on-call services to be experts and those with <5 years of work experience as novices based on information from previous studies.^[21,22]^ We utilised several means including snowballing sampling technique to reach as many physiotherapists as possible to participate in the present study.

We found that 197 items of knowledge and skills were judged to be ‘essential’ as a minimum standard of clinical practice in critical care settings following three rounds of online Delphi survey. Consensus was not reached to classify any question item from the questionnaire as ‘not essential’ for clinical practice. The results of the present study are similar to those of Takahashi *et al*.^[12]^ who reported consensus for 199 items from a partially modified version of the Skinner *et al*.^[10]^ questionnaire. Nevertheless, our findings are different from those in Australia^[10]^ and the UK,^[11]^ where consensus was reached for fewer items as ‘essential’ and ‘not essential’. We think that the experts in our study had comparatively lower experience and exposure in ICUs as they were more likely to have received little or no training, and they were practicing in a resourcelimited setting. It is important to note that the scope of practice of physiotherapists in the ICU differ across countries.^[21,23]^

We had a good response rate, considering the low number of physiotherapists with experience in ICU clinical practice in Nigeria. Our study also recorded very low dropout rates between rounds. Twose *et al*.^[11]^ reported significantly higher dropout rates in their study (from 80% in round 1 to 65% in the final round). However, this is still within the accepted range for Delphi studies.^[24]^ It must also be stated that several reminder messages were sent to non-responders via phone calls in addition to emphasising the need to observe the deadline.^[25]^ In addition, sending the questionnaire via WhatsApp was helpful as most participants had smartphones. Overall, the number of participants in our study was small compared with other studies, but we considered them as having the best critical care experience in view of the strict inclusion criteria.

Participants in the present study ranked more items as ‘essential’, and no items as ‘non-essential’, unlike other studies that recorded several items from the same questionnaire (Australia,^[10]^ New Zealand,^[10]^ Japan^[12]^ and UK^[11]^). Van Aswegen *et al*.^[26]^ reported consensus was achieved on knowledge of normal integrated anatomy and physiology, knowledge of and skill to conduct a holistic assessment of an ICU patient, knowledge and skill of clinical reasoning, and knowledge of physiotherapy techniques by physiotherapists working in critical care units in South Africa. Another reason for the high number of ‘essential’ question items reaching a definite consensus after the final round was because we modified the questions not reaching consensus by providing definitions and examples during the second and third rounds. Therefore, the additional consensus obtained for these items that were modified could mean that our study participants may not have been conversant with some of the question items as presented in the Skinner *et al*.^[10]^ questionnaire. Specifically, most of the items not reaching consensus appear to require intensive training and high clinical skills. Therefore, it is not surprising that items on intubation/extubation of patients, interpreting measurements in ICUs such as haemofiltration, pulmonary arterial catheter measurements, C-reactive protein as well as performing cuff volume and/or pressure test on an endotracheal tube or tracheostomy, among others, did not reach consensus.

The skills and knowledge that a physiotherapist needs to deliver services independently in critical care need to be streamlined.^[27]^ Moreover, there is lack of standardised programmes for ICU physiotherapist education in our environment.^[28]^ In the present Delphi study, the participants appeared to agree with the majority of the items on the questionnaire, with no tangible additional items suggested across the rounds. This may be because the requirements for practice are less strict in terms of scope and responsibilities of physiotherapists in Nigeria compared with many advanced countries.^[29,30]^ We also observed that some question items such as the Feldenkrais technique, which is not common in our critical care settings, reached consensus after the third round, after alternative definitions and examples were provided. Furthermore, items like bronchial lavage, which is a diagnostic method of the lower respiratory system in which a bronchoscope is passed into the lungs with a measured amount of fluid introduced and then collected for examination, reached consensus in the first round without necessitating alternative definitions. This technique is typically outside the physiotherapist’s scope of practice, not popular in our environment and requires high technological and technical expertise. Hence, we cannot at the moment explain why the participants must have checked ‘essential’ for this item. Nevertheless, it is important that we could still draw attention to the consensus opinion of participants of the present study using this kind of question items. Moreover, more physiotherapists working in the critical care setting are increasingly having the opportunity to acquire further knowledge and skills from online ICU rehabilitation workshops delivered by people from all parts of the world. Our study offers a good first step in approaching institutions with possible items that could form a curriculum for training of physiotherapists who will work in ICUs in Nigeria. The existing curriculum in undergraduate physiotherapy programmes in Nigeria does not provide for teaching and clinical practice experience in most of the items checked as ‘essential’. Therefore, the use of graduate physiotherapists without additional specialised training in the ICU at postgraduate level may not be desirable.

### Study limitations

The instrument from Skinner *et al*.^[10]^ was not locally validated prior to administration in the present study. It was noted that the questionnaire was quite detailed, with many question items covering all aspects of critical care. Nevertheless, the study results are valid because we used a consensus to arrive at the items selected eventually. Participants were given a chance in the first round of the Delphi to provide additional inputs to the question items by means of open-ended questions requiring them to suggest additional questions. However, no further suggestions were made and this may mean that they were satisfied with the instrument or they had response exhaustion due to the many questions, as observed in previous Delphi studies.^[31]^ In the present study, the questionnaire comprised many questions and required ~30 minutes to complete. Hence, participants were given up to 2 weeks to complete the questionnaire for each round. The critical care capacity, resources and manpower are relatively low and limited in Nigeria compared with developed countries,^[32]^ so the number of physiotherapists who were available for recruitment was relatively small. Future studies should focus on testing the knowledge of physiotherapists working in the ICU contained in the ‘essential items’ reaching consensus with a view to ascertaining specific areas of need in this environment.

## Conclusion

Expert consensus was achieved for a substantial number of questions on knowledge/skills of assessment, condition and treatment. These items could be considered as ‘essential’ minimum standards of clinical practice for physiotherapists working in ICUs, based on the opinion of experienced physiotherapists in Nigeria. The results of this present study need to be further validated by appropriate authorities to support development of training programmes and curricula for critical care physiotherapy specialisation in Nigeria with a view to reducing clinical practice variability and achieving acceptable quality in patient management.
